# Voyages in Vacation.—VIII

**Published:** 1888-11-17

**Authors:** 


### Voyages in Vacation.—VIII.

By One in Search of Restful Restoratives.

(*Continued from page* 87.)

### CRONSTADT AND ST. PETERSBURG FROM THE SEA.

The glimpses obtained of the coasts of Finland and Russia from the steamer after land is sighted in the Baltic, and as the vessel approaches Cronstadt, are not attractive. Low-lying, flat, uncultivated, and barren, the country appears to be one of anything but promise to the traveller. Within a few miles of Cronstadt we met a Russian ironclad, and as we passed very close to it we were able to make out the vessel distinctly, and to admire the picturesque costumes worn by the officers and men, whose white caps were specially attractive and becoming. The channel becomes more difficult as the town of Cronstadt is neared and the fortifications are observed. These forts are unusually strong and extensive, and the chief of them are built on rocks or sunken dams which block the northern channel, and has no less than seven batteries upon it. The forts present very little surface to aim at, and it would no doubt prove nearly, if not quite, impossible to take them by assault. Indeed, so little of them is plainly visible from the Baltic that they present the appearance of being relatively small and unimportant when examined through a glass. Far from this being in reality the case, it became evident that they are surprisingly large when we had passed them and cast anchor off Cronstadt harbour. The outer defences, of which the southern appears to be the strongest, consist of three lines of forts, running from west to east. To the south and north-west are two batteries and two redoubts. The land defences are also formidable, being arranged in parallel lines, and connected by earthworks and batteries. To our surprise, a considerable force of troops were encamped behind some of the forts which the Russians have good reason for believing to be impregnable, when the difficulties of the channel is borne in mind. Cronstadt has very extensive harbours and docks, where many vessels of considerable size were unloading or taking in cargo. Two-thirds of the merchant vessels, which average some twelve to fifteen hundred annually, are English, and they are accommodated in two harbours near the town. This was our first experience of a Russian town, and the many-coloured roofs, with the gilded domes of the mosques, gave a very brilliant appearance to the place when seen, as we saw it, to the best advantage, owing to the brilliant sunshine which welcomed us on our arrival. The painted roofs and resplendent domes gave quite a novel and cheerful appearance to all Russian cities, and make them as unique as, in bright weather, they are undoubtedly brilliant in appearance.

We had understood that there would be a considerable delay at Cronstadt, owing to the necessity for passing the Customs and quarantine officers. No sooner had we cast anchor than a steam launch boarded the *Victoria*, and placed two officers in charge of the steamer to prevent anyone from leaving for the shore or bringing anything on board. These Russian officials took entire possession of the vessel, and during the whole of our stay at St. Petersburg they never left the ship. Time seems to be no object to the Russian officials, and so we had to wait a considerable time before further notice was taken of the *Victoria* or any serious attempt was made to inspect the passengers and crew, so as to grant us permission to proceed through the canal into the Neva. When the authorities at last came, they proved to be so numerous as to constitute quite a ship's company. First came the quarantine officer and his staff, then the harbour authorities, and last of all the Customs. The crew were summoned to the quarter-deck, where all hands were closely inspected, the Russians examining each man and woman very closely, to our no small amusement. Our time had yet to come, for no sooner were the passengers comfortably seated at luncheon than an order came for all of them to proceed on deck to enable the authorities to satisfy themselves from personal observation that there was not a Nihilist amongst us. How they were supposed to know this we did not ascertain until afterwards. It was noticed, however, that several flat tin cases were brought on board which were supposed to contain books for entries, etc. It turned out that some of them contained photographs of suspicious and dangerous characters who are *anathema* so far as Russia's shores are concerned. It was the duty of one of the officers, as representing the Emperor, to satisfy himself that no one resembling any of the persons photographed was permitted to set foot in St. Petersburg. Hence the mustering and examination of passengers and crew, and their individual inspection. Passports were next sent for, but these gave us no trouble, as the purser took charge of them, and returned them, duly viséd, after the Russian officers had departed. Of course all this took a good deal of time, and whilst it was going on, and when the passengers and crew were on deck, some of the Russians were making a visit to every part of the ship, including cabins and the officers' and crews' quarters, so as to satisfy themselves that no one was in hiding, and that no contraband goods were stowed away in out-of-the-way places. This finished, the Russian officials adjourned to the saloon and partook of refreshment, it being noticeable that whatever they might individually eat their powers of imbibing were of a rare order, and the facility with which some of these gentlemen could "mix their liquors" excited wonder or admiration, according to the views held on the temperance question by those who witnessed it. The Russians proved to be very polite, and they succeeded in discharging duties which must at times prove very irksome, in a pleasant and fairly expeditious manner. We were not sorry, however, when the pilot came on board, and a tug arrived to take us through the canal.

The canal which connects Cronstadt roads with the mouth of the river Neva at Gutuéf Island was commenced in 1877, and finished about 1885. It is nearly 26 versts in length, from 210 to 280 feet in breadth in the widest part—*i.e.*, at the St. Petersburg end—but it is only 125 feet in width for the first 16 versts. Its depth in the centre is kept, by dredging, at never less than 22 feet. Its object is to enable merchant vessels to load and unload at St. Petersburg, so that Cronstadt may be reserved as a purely naval dockyard and harbour. The canal was rendered necessary by the narrowness of the Neva, near the bar, at its mouth, where it is seldom, if ever, more than nine feet in depth. Anyone who has traversed the canal will be struck by the engineering and other difficulties which have been so successfully surmounted. At the junction of the canal with the Neva, the pilot points out the place where the *Ceylon* was run hard aground two years ago, she being unlucky enough to drag her anchors subsequently, and so to foul the Nicholas Bridge. Some allowance must be made for local colouring, but so far as we could ascertain, both these accidents happened. They may be held to show that large ocean-going steamers should not attempt to pass quickly through the canal or Neva, and that anchorage should be found far enough from the bridge to avoid unpleasant risks of the latter kind.

As the Neva was entered, the domes of St. Isaac, the Kazan, and St. Peter and St. Paul cathedrals were conspicuous and striking. It is, no doubt, a common belief amongst English people that Russia is a poor country, possessing comparatively little trade and few, if any, manufactories. A stranger is not prepared, therefore, to see the signs of active, continuous, and extending manufactories, the bustle and stir of many workpeople busily engaged in profitable trades, the numerous and extensive warehouses and manufactories, in addition to innumerable private and Government shipbuilding yards which confront the eye as the steamer proceeds from Gutuéf Island to St. Petersburg. Volumes of smoke, numerous steamers plying hither and thither, and other signs of prosperity and business enterprise soon disillusionise the visitor, who soon realises that Russia is not, after all, so much behind other nations, at any rate at St. Petersburg.

*(To be continued.)*

